# Sigmoid Perforation by an Ingested Foreign Body Mimicking Acute Appendicitis: A Case Report

**DOI:** 10.7759/cureus.66855

**Published:** 2024-08-14

**Authors:** Nithya Devanathan, Hemangi Patel, Pinar Sargin, Alberto Zarak, Alejandro Biglione

**Affiliations:** 1 Medical School, Nova Southeastern University Dr. Kiran C. Patel College of Osteopathic Medicine, Fort Lauderdale, USA; 2 Sports Medicine, Nova Southeastern University Dr. Kiran C. Patel College of Osteopathic Medicine, Fort Lauderdale, USA; 3 Internal Medicine, Wellington Regional Medical Center, Wellington, USA; 4 Surgery, Wellington Regional Medical Center, Wellington, USA

**Keywords:** abdominal infection, acute abdomen, foreign body ingestion treatment, foreign body ingestion complications, sigmoid perforation

## Abstract

Lower abdominal pain is a common complaint for patients presenting for evaluation in the emergency department. Among other life-threatening complications, acute appendicitis needs to be ruled out in the case of right-lower quadrant pain (RLQ). Sigmoid perforation caused by an ingested foreign body is an uncommon cause of RLQ pain. This report presents the case of an otherwise healthy, 29-year-old male who presented to the emergency department with RLQ pain. His initial evaluation raised concern for acute appendicitis. However, during his exploratory laparoscopy, he was found to have a perforated sigmoid colon due to a skewer stick; the patient had no recollection of having ingested any foreign body. This paper highlights the importance of considering the possibility of sigmoid perforation by an ingested foreign body as a possible cause of RLQ pain. The article also reviews the most common causes of ingested foreign bodies, their potential complications and management.

## Introduction

Intestinal perforation is the loss of continuity of bowel structures due to trauma, infection, malignancy, or ischemia [1]. Perforation can occur when sharp foreign objects within the gastrointestinal tract penetrate the bowel wall. Ingested foreign bodies can pass through the esophagus and stomach without causing any complications, however, they can cause obstruction when they transit through the bowel. It is important to note that patients are not always aware that they have swallowed a foreign object [1,2]. We present the case of a 29-year-old male who presented to the emergency department with lower abdominal pain and underwent an exploratory laparotomy for possible appendicitis but was found to have a perforated sigmoid bowel caused by a kebab skewer. The patient does not recall when and how the foreign body was ingested.

## Case presentation

The patient is a 29-year-old male who presented to the emergency department with abdominal pain. He described the pain as sharp in character, located in the lower abdomen, and severe in intensity that had started two days before presentation. He denied any associated nausea, vomiting, diarrhea, hematemesis, or melena. 

The patient's initial vital signs in the emergency room were: temperature of 98.9 ºF (37.2 ºC), heart rate of 82 beats per minute, blood pressure of 144/94 mmHg, respiratory rate of 14 respirations per minute, and oxygen saturation of 98% on room air. On physical examination, the chest was clear to auscultation. The abdomen did not show any abnormalities on inspection. On palpation, the abdomen was soft and tender to deep palpation over the right-lower quadrant (RLQ) and hypogastrium with no guarding. There were diffuse decreased bowel sounds on auscultation.

The patient's labs were completed and the pertinent lab values can be found below in Table 1. 

**Table 1:** Lab values of the patient with corresponding normal ranges.

Parameters	Patient Values	Normal Range
Sodium	135 mmol/L	136-146 mEq/L
Potassium	4.0 mmol/L	3.5-5.0 mEq/L
Carbon dioxide	29.0 mEq/L	23-29 mEq/L
BUN (blood urea nitrogen)	13 mg/dL	7-18 mg/dL
Creatinine	0.83 mg/dL	0.6-1.2 mg/dL
Alkaline phosphatase	52 IU/L	25-100 IU/L
AST (aspartate transaminase)	37 IU/L	12-38 IU/L
ALT (alanine aminotransferase)	74 IU/L	10-40 IU/L
Bilirubin	0.4 mg/dL	0.1-1.0 mg/dL
WBC (white blood count)	12.69 x 10^3^/mcL	4.5 x 10^3^/mcL - 11 x 10^3^/mcL
RBC (red blood count)	5.9 x 10^6^/mcL	4.35 x 10^6^/mcL – 5.65 x 10^6^/mcL
Hemoglobin	14.8 g/dL	14-16 g/dL
Hematocrit	44.7%	40-54%
Platelet count	253 x 10^3^/mcL	150 x 10^3^/mcL - 450 x 10^3^/mcL
Lipase	79 IU/L	0-160 IU/L

**Figure 1:**
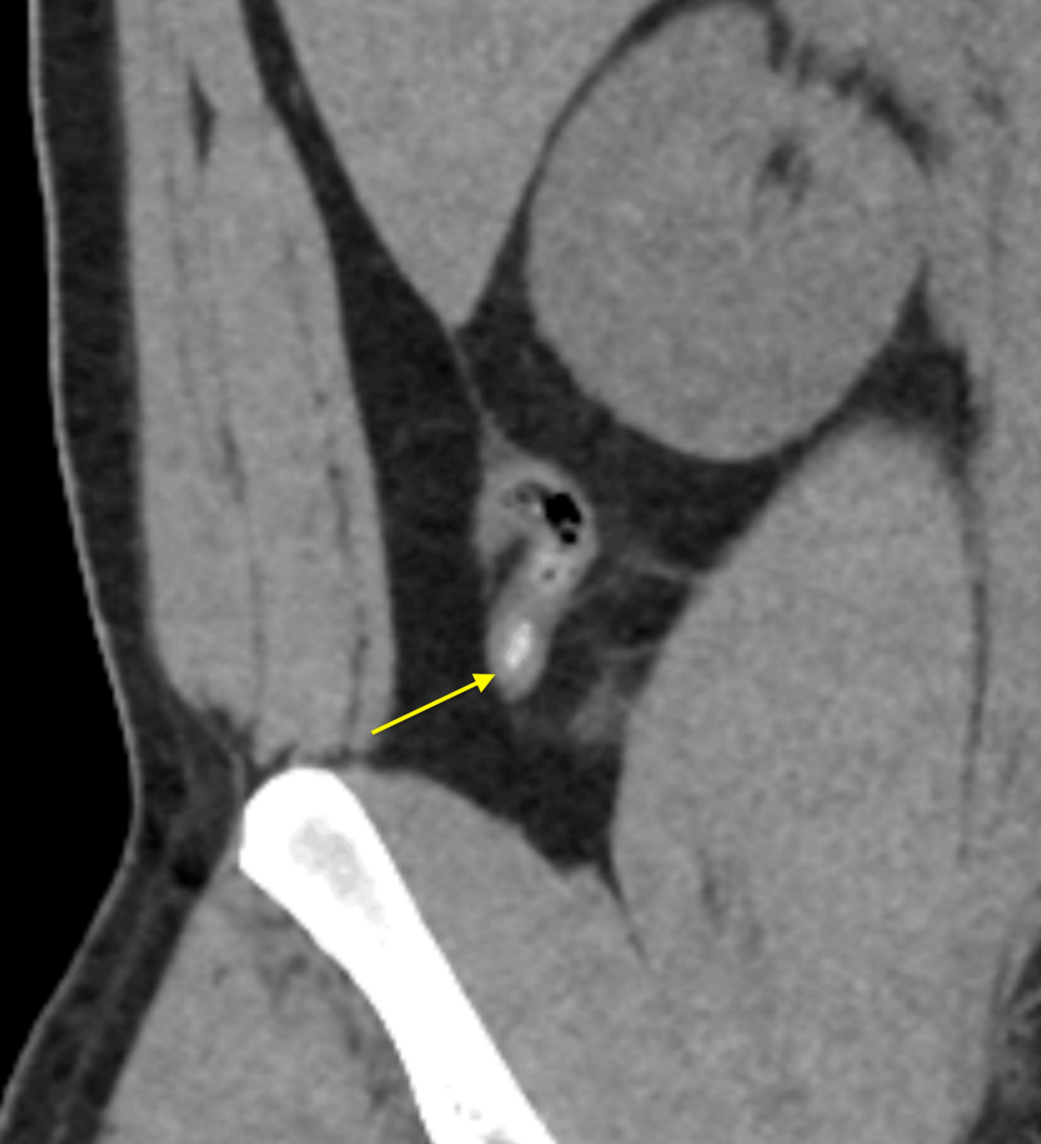
CT abdomen/pelvis without contrast showing a mildly thickened appendix measuring 8 mm without signs of perforation (yellow arrow).

**Figure 2:**
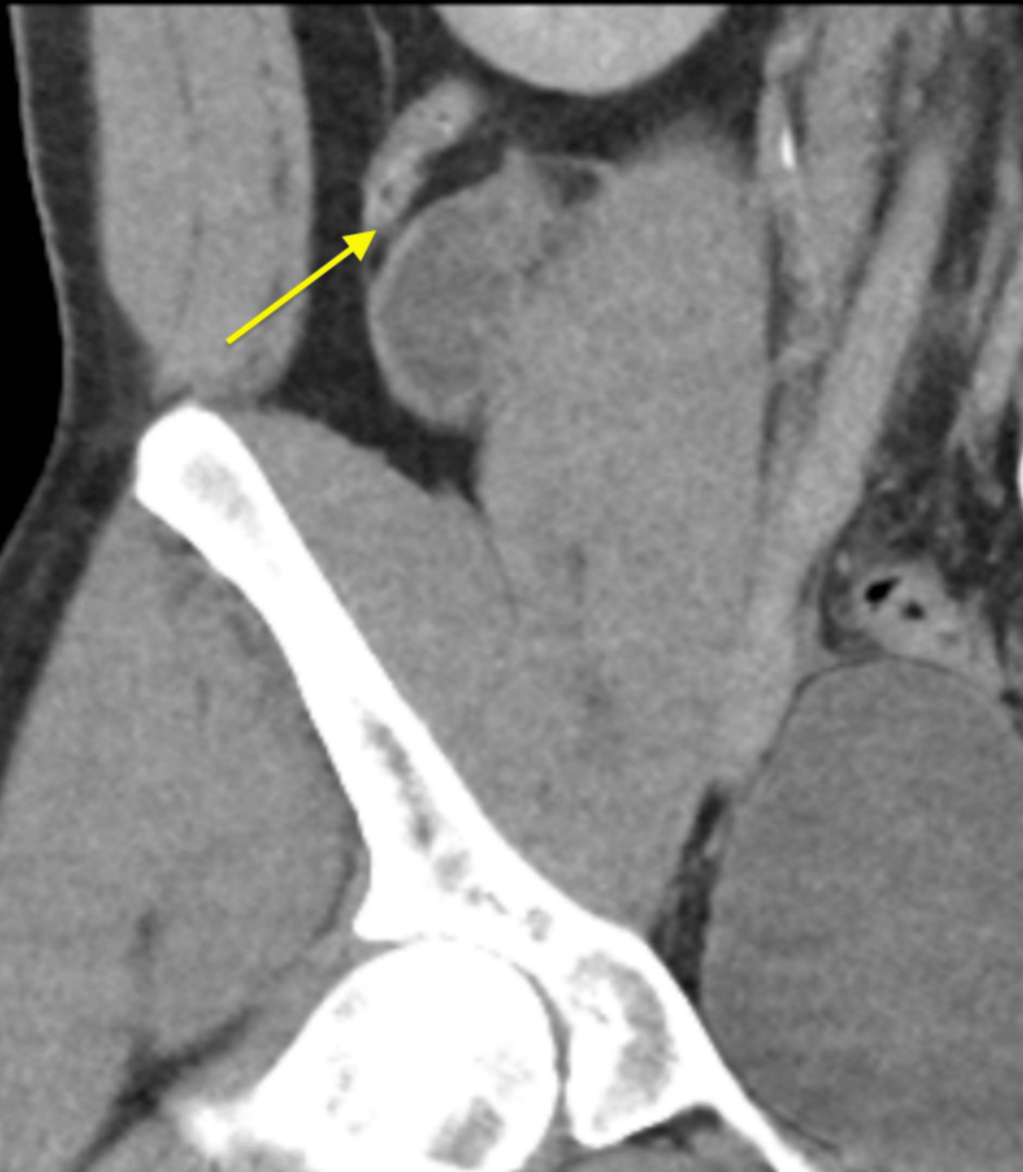
CT abdomen/pelvis with contrast showing a mildly thickened appendix measuring 8 mm without signs of perforation, similar to the imaging without contrast (yellow arrow).

An initial non-contrast CT scan of the abdomen and pelvis was performed and showed a normal liver, pancreas, adrenal glands, kidney, and spleen. The gallbladder had normal wall thickness and no apparent calculi. The stomach, small bowel, and mesentery were unremarkable. The appendix was enlarged, with a diameter of 8 mm (Figures 1-2). The normal size of the appendix is less than 6 mm. There was no abdominal mass or fluid collection present. The patient was admitted for further management and the general surgery team was consulted.

**Figure 3:**
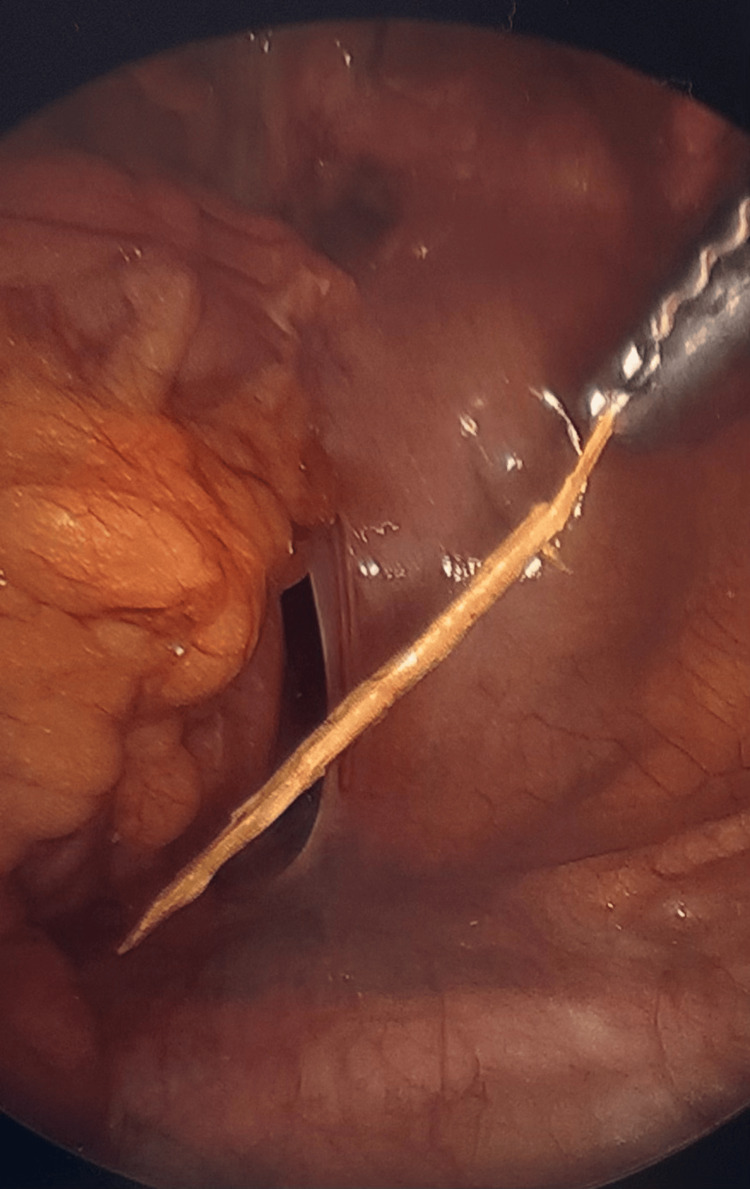
Trans-laparoscopic procedural view. Photograph visualizing the foreign body being removed from the sigmoid colon.

**Figure 4:**
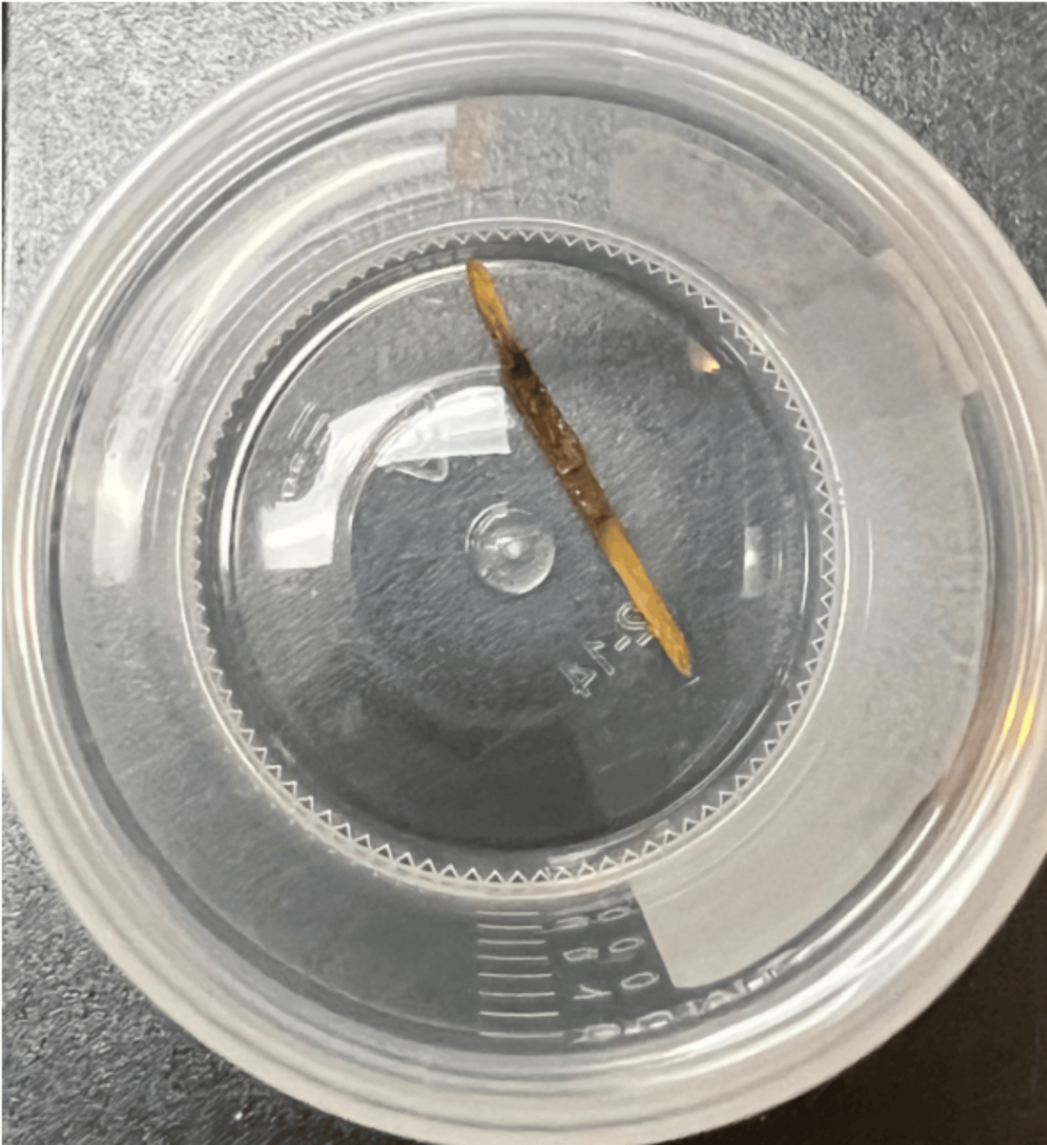
Foreign body specimen retrieved in surgery.

The following morning, the patient was scheduled for a laparoscopic appendectomy. While in the operating room, the surgeon immediately noticed purulent fluid in the pelvis. The purulent fluid within the abdomen was indicative of peritonitis. A section of the sigmoid colon was severely inflamed and a foreign body was found sticking out of the colonic wall. The foreign body was tan, brown, 3.4 cm by 0.2 cm. The object was retrieved by the laparoscopic instruments. Upon removal of the object from the sigmoid colon, it was found to resemble a kebab skewer (Figures 3-4). After the foreign body was removed, the surgeon continued with the laparoscopic appendectomy due to the presence of the thickened and enlarged appendix on CT imaging. 

**Figure 5:**
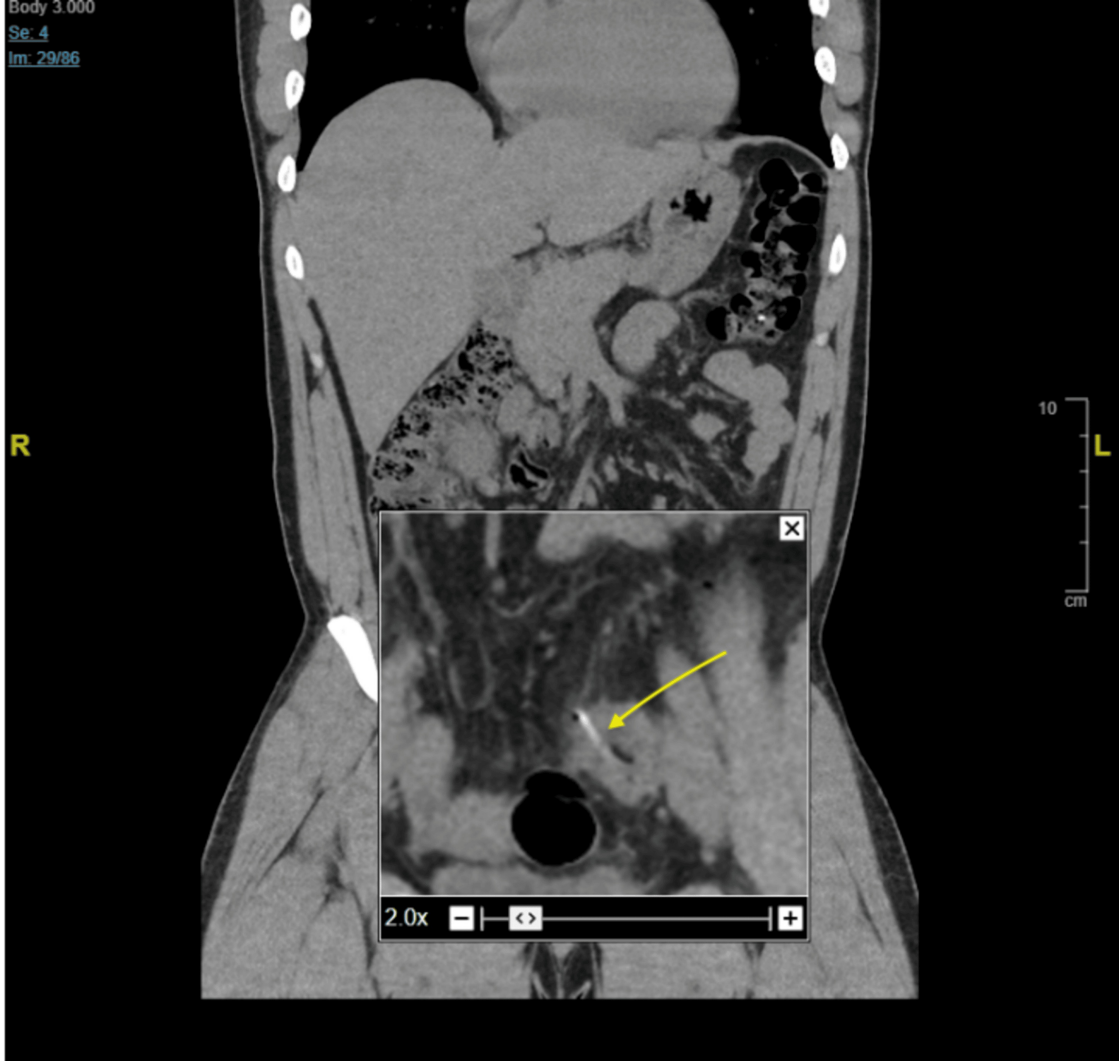
CT abdomen/pelvis without contrast showing a radio-opaque foreign object in the sigmoid colon perforating the bowel wall visualized by the yellow arrow.

Based on the intraoperative findings, the attending physician reviewed the initial non-contrast CT and identified the perforating foreign object (Figure 5). The patient’s postoperative period was uneventful. He received IV cefepime and metronidazole for the treatment of peritonitis. The patient initially received clear liquids and was advanced to full liquids and a soft mechanical diet.

The patient was discharged and told to follow up with general surgery and his primary care physician. He was advised to call the surgeon if he developed any fever, chills, abdominal pain, nausea, vomiting, bloody stools, or drainage from the laparoscopic ports. He was discharged on amoxicillin and ciprofloxacin for a 10-day antibiotic treatment to prevent postoperative wound infections. He was advised to follow a low-fiber and low-residue diet. 

## Discussion

Some of the most commonly ingested foreign bodies include fish bones, bones from other meat sources, and dentures [3]. Accidentally swallowing foreign objects with food is the most common cause of ingested foreign bodies in adults [3]. Patients will present with symptoms including acute lower abdominal pain, fever, vomiting, nausea, melena, and hematochezia [4]. Often, the patient can also present with RLQ pain with the previously mentioned symptoms, mimicking the gastrointestinal diagnosis of appendicitis [4]. Most foreign bodies pass through the gastrointestinal tract without complications. The most common life-threatening complications that arise are internal bleeding, perforation, and obstruction [5]. However, only 10-20% of foreign body ingestion cases result in surgical intervention [5]. 

In this case, the patient presented with similar symptoms of appendicitis which prompted an immediate laparoscopic procedure. However, only upon laparoscopic intervention were the findings of peritonitis and sigmoid perforation caused by a foreign body discovered. The perforation of the sigmoid colon occurs in less than 1% of cases, making the findings of this case a rare occurrence [5]. Many complications can arise from foreign body ingestion including perforation and secondary peritonitis. Laparoscopic removal of foreign bodies from the peritoneal cavity tends to lead to small bowel perforations [6]. Small bowel perforations are treated with a lavage of the abdominal cavity and closure of the perforation, usually with an omentoplasty [6]. This procedure is more likely indicated when there is an irregular foreign body such as a safety pin or a large foreign body like a fork. In the patient presented, the kebab skewer was small in size with a smooth contour, thus, eliminating the need for a colectomy. 

The diagnosis was challenging due to the patient's inability to remember ingesting the offending foreign object and conflicting symptoms on patient presentation. Radiologists can miss ingested foreign bodies in conventional radiographic imaging, such as X-rays, due to the presence of fluids and soft tissue. Multi-slice CT scan imaging is the preferred choice in such cases due to the ability to visualize an object's size, shape, or radiodensity [3]. However, even with the completion of CT imaging on this patient, the offending foreign object was not mentioned in the initial radiology report. The attending physician later identified the foreign body on the initial CT scan after the surgery (Figure 2).

## Conclusions

As examined in this report, a foreign body ingestion led to abdominal pain mimicking symptoms of acute appendicitis with RLQ pain. However, upon proper evaluation, it was determined that the patient had a sigmoid bowel perforation with signs of acute peritonitis. This paper intends to raise awareness of these unusual clinical diagnoses in the setting of a common presentation of acute RLQ pain.
